# Publisher Correction: Evolutionary adaptations to new environments generally reverse plastic phenotypic changes

**DOI:** 10.1038/s41467-018-03360-3

**Published:** 2018-02-21

**Authors:** Wei-Chin Ho, Jianzhi Zhang

**Affiliations:** 1 Department of Ecology and Evolutionary Biology, University of Michigan, Ann Arbor, MI 48109 USA; 2 Present Address: Center for Mechanisms of Evolution, The Biodesign Institute, Arizona State University, Tempe, AZ 85287 USA

Correction to: *Nature Communications* 10.1038/s41467-017-02724-5, published online: 24 January 2018

The originally published HTML version of this Article contained errors in the three equations in the Methods sub-section ‘Metabolic network analysis’, whereby the Greek letter eta (η) was inadvertently used in place of beta (β) during the production process. These errors have now been corrected in the HTML version of the Article; the PDF was correct at the time of publication.

