# Supplementary material for: Evolutionary adaptations to new environments generally reverse plastic phenotypic changes
Source: Nat Commun. 2018 Jan 24;9:350. doi: 10.1038/s41467-017-02724-5 (PMC5783951; doi:10.1038/s41467-017-02724-5)
Supplement: Supplementary file 1 — Supplementary Information [file 41467_2017_2724_MOESM1_ESM.pdf]

Supplementary materials for

Evolutionary adaptations to new environments generally reverse plastic phenotypic changes

Ho & Zhang (jianzhi@umich.edu)

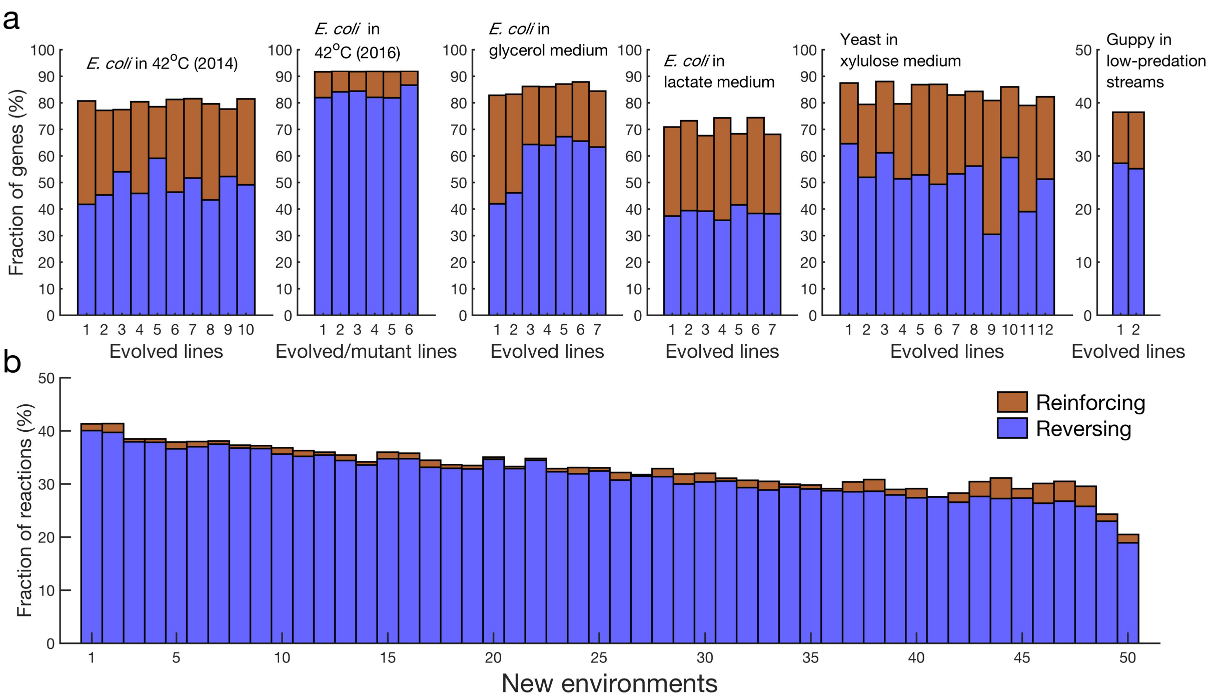

Supplementary Fig. 1. Genetic adaptations more frequently reverse than reinforce plastic phenotypic changes. The cutoff of $0.05L_o$ is used in defining plastic and genetic changes. (*a*) Fractions of genes with reinforcing and reversing expression changes, respectively, in experimental evolution. Organisms as well as the new environments to which the organisms were adapting to are indicated. Each bar represents an adaptation. (*b*) Fractions of reactions with predicted reinforcing and reversing flux changes, respectively, in *E. coli*'s adaptations to 50 new environments from the glucose environment.

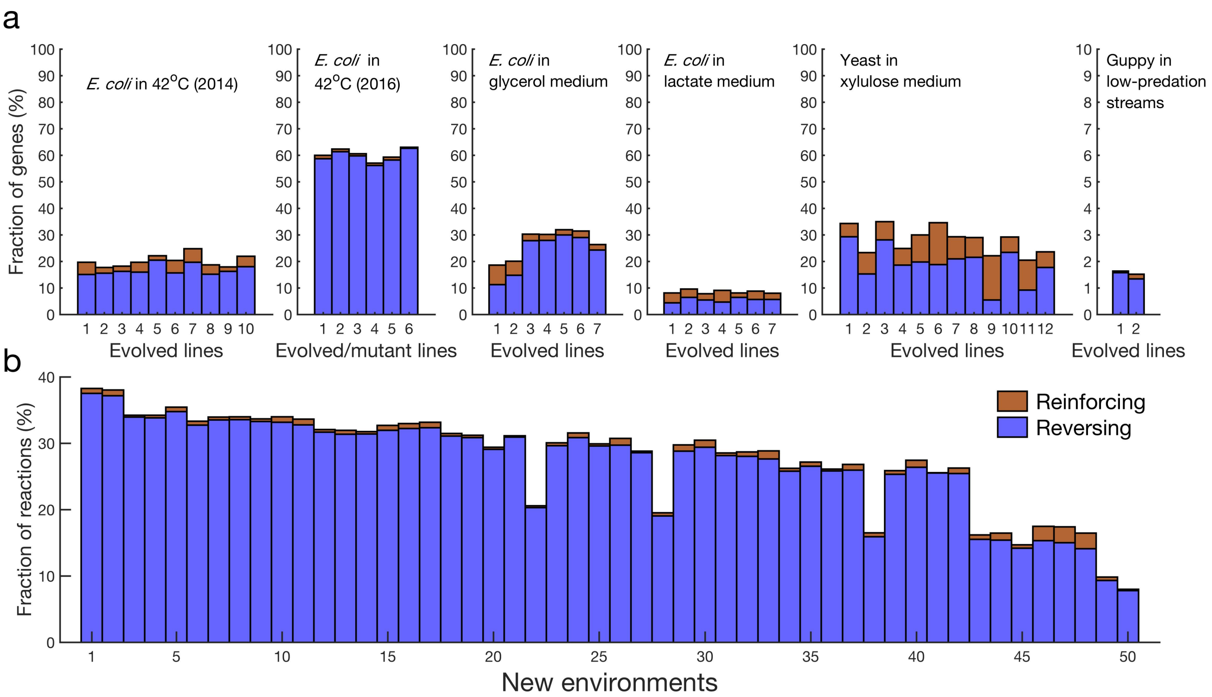

Supplementary Fig. 2. Genetic adaptations more frequently reverse than reinforce plastic phenotypic changes. The cutoff of $0.5L_o$ is used in defining plastic and genetic changes. (*A*) Fractions of genes with reinforcing and reversing expression changes, respectively, in experimental evolution. Organisms as well as the new environments to which the organisms were adapting to are indicated. Each bar represents an adaptation. (*B*) Fractions of reactions with predicted reinforcing and reversing flux changes, respectively, in *E. coli*'s adaptations to 50 new environments from the glucose environment.

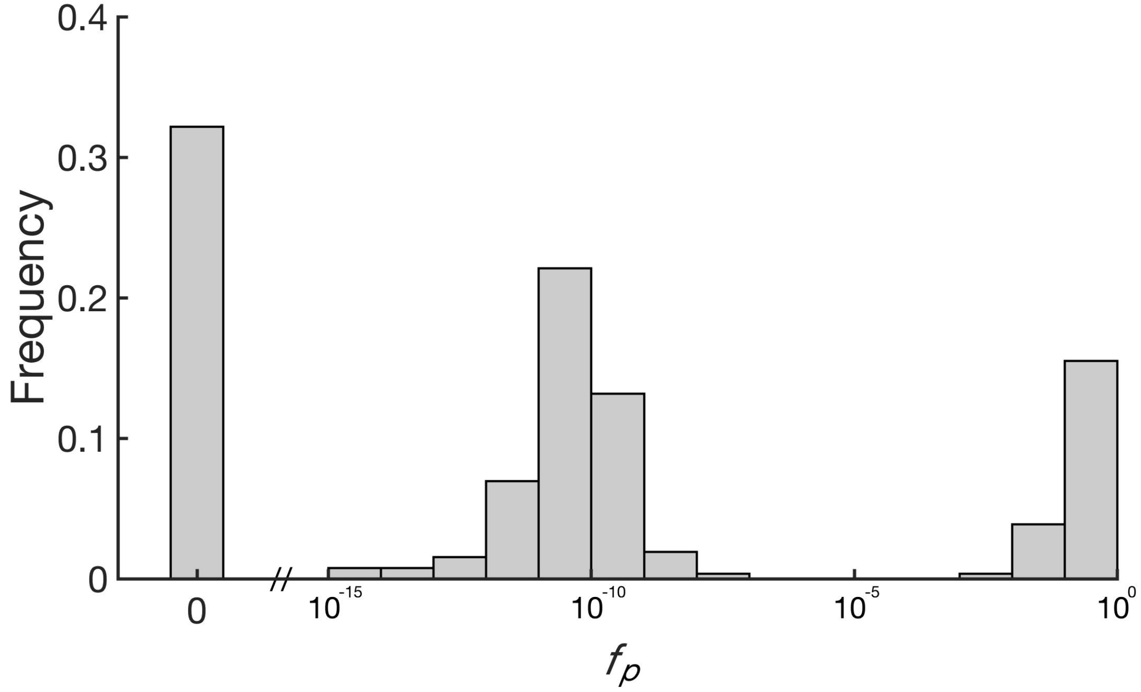

Supplementary Fig. 3. Frequency distribution of the fitness of *E. coli i*AF1260 at the plastic stage (f_p) in 257 new environments relative to that in the original glucose environment.

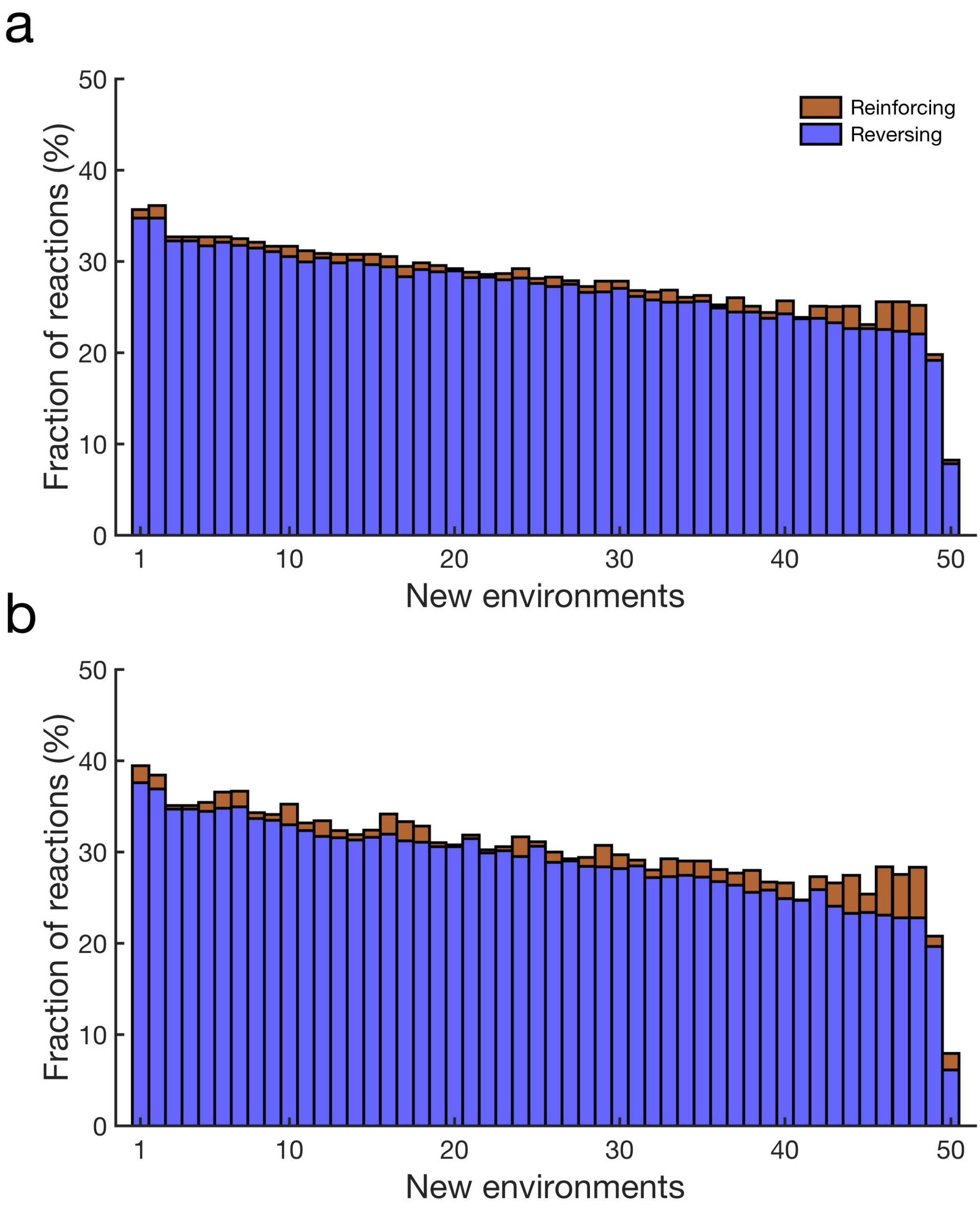

Supplementary Fig. 4. Predominance of flux reversion in the adaptations of *E. coli* iAF1260 to 50 new environments when the metabolic analysis uses (*a*) a randomized order of reactions in the stoichiometric matrix or (*b*) MOMA-b to predict fluxes at stage *a*.

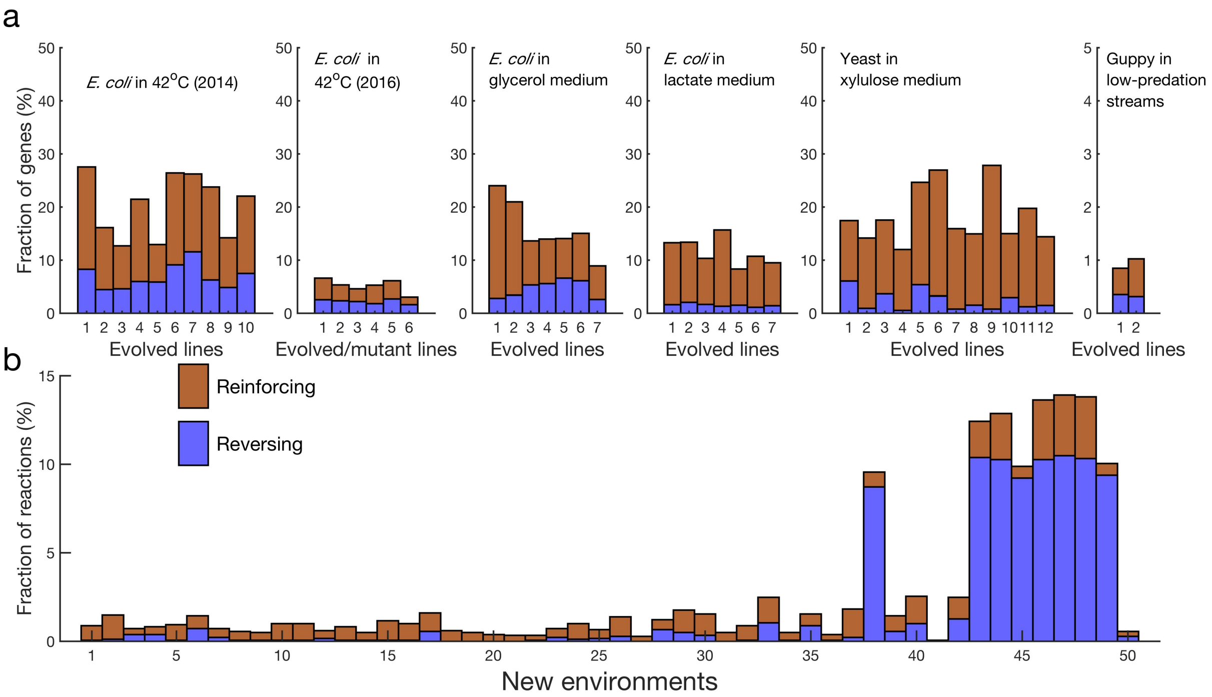

Supplementary Fig. 5. The preponderance of phenotypic reversion disappears after the removal of traits for which the size of the plastic change exceeds that of the total change (*PC* > *TC*). (*a*) Fractions of genes with reinforcing and reversing expression changes, respectively, in experimental evolution. Organisms as well as the new environments to which the organisms were adapting to are indicated. Each bar represents an adaptation. (*b*) Fractions of reactions with predicted reinforcing and reversing flux changes, respectively, in *E. coli*'s adaptations to 50 new environments from the glucose environment.

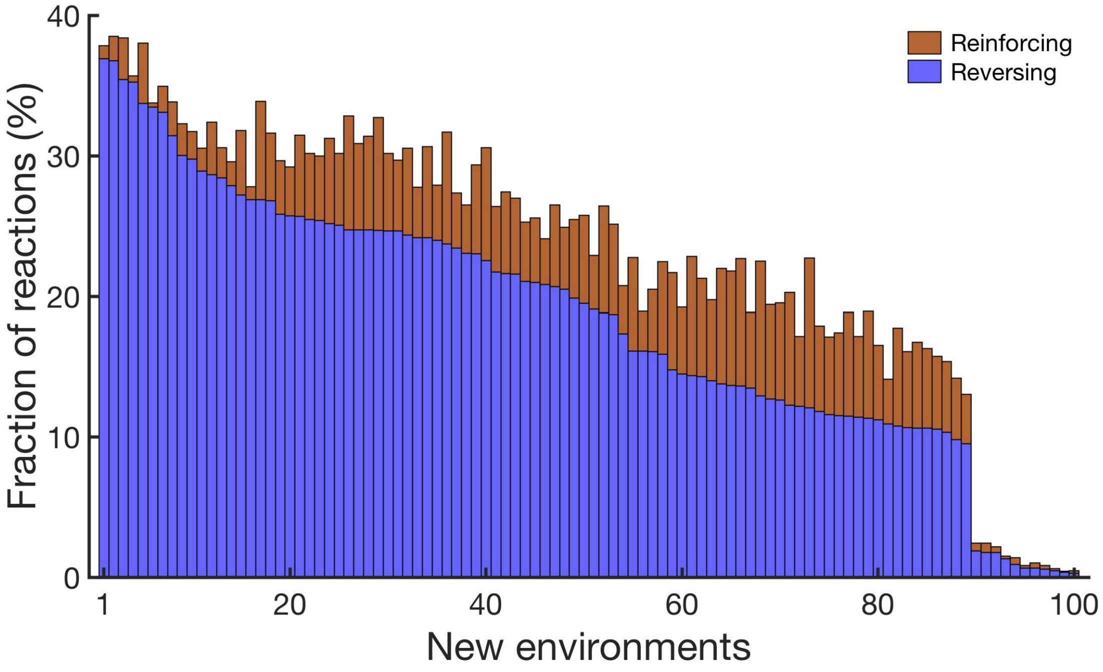

Supplementary Fig. 6. Predominance of flux reversion in the adaptations of *E. coli* iAF1260 to 100 new complex environments from the glucose environment. Each complex environment contains multiple carbon sources (see Methods).

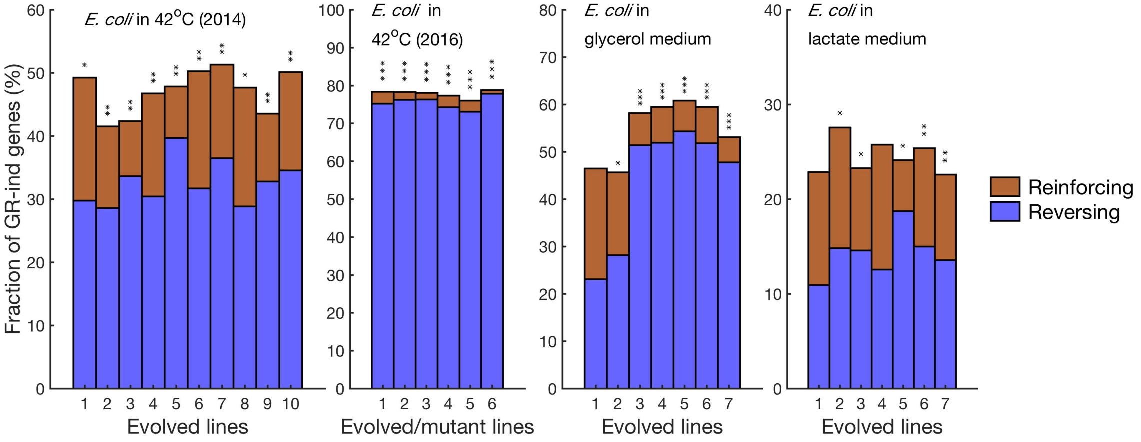

Supplementary Fig. 7. Genetic adaptations of *E. coli* in experimental evolution more frequently reverse than reinforce plastic gene expression changes for growth-rate-independent (GR-ind) genes. Shown are fractions with reinforcing (C_{RI}) and reversing (C_{RV}) expression changes, respectively. Organisms as well as the new environments to which the organisms were adapting to are indicated. Each bar represents an adaptation. The equality in the fraction of reinforcing and reversing genes in each adaptation is tested by a two-tailed binomial test. When $C_{\mathrm{RV}} > C_{\mathrm{RI}}$, *P*-values are indicated as follows: *, $P < 0.05$; **, $P < 10^{-10}$; ***, $P < 10^{-100}$; otherwise, *P*-values are indicated as follows: °, $P < 0.05$; °°, $P < 10^{-10}$; °°°, $P < 10^{-100}$.

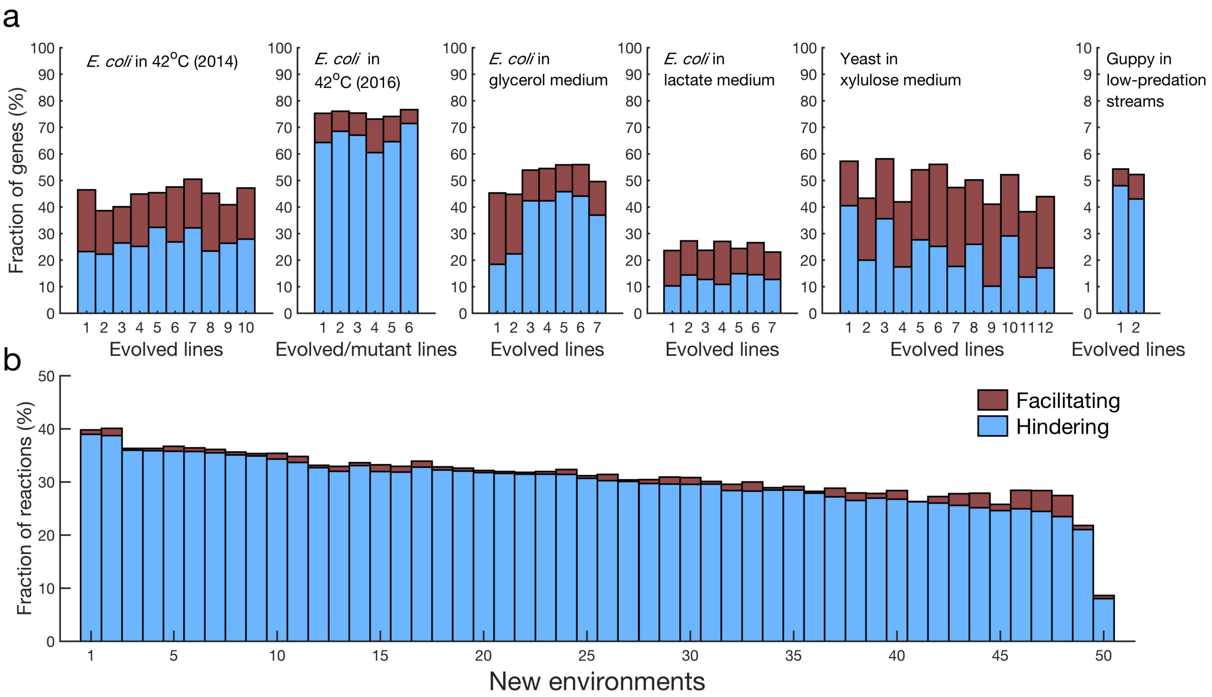

Supplementary Fig. 8. Facilitating plasticity is generally less prevalent than hindering plasticity in adaptations. See main text for definitions of facilitating and hindering plasticity. (*a*) Fractions of genes showing facilitating and hindering expression plasticity, respectively, in each of the 44 cases of experimental evolution. (*b*) Fractions of reactions showing facilitating and hindering flux plasticity, respectively, in each of the 50 environmental adaptations of the *E. coli* metabolic network from the glucose medium.

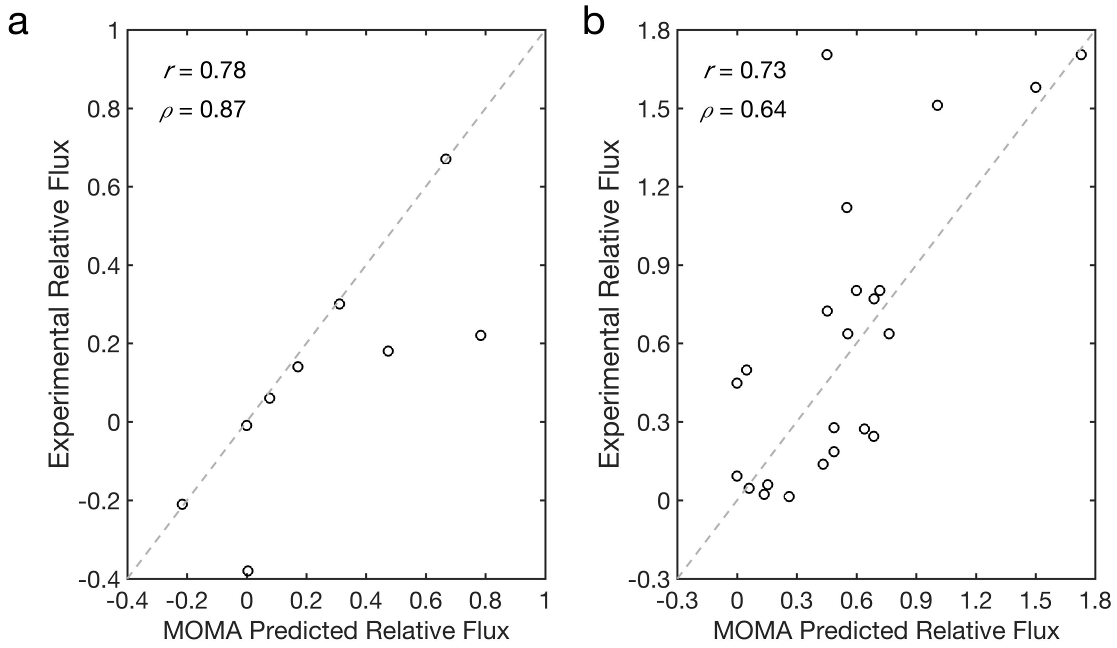

Supplementary Fig. 9. Correlation between relative fluxes experimentally measured and fluxes predicted by minimization of metabolic adjustment (MOMA). All fluxes are relative to the uptake rate of the respective carbon source. Each dot represents the flux of a reaction. *r*: Pearson's correlation coefficient; *ρ*: Spearman's correlation coefficient. (*a*) Fluxes were measured and predicted in the lactate environment. (*b*) Fluxes were measured and predicted in the galactose environment.

Supplementary Table 1. The 50 single-carbon source environments to which the *E. coli* metabolic network can adapt to from the glucose environment

Number	Abbreviaiton	Full name
1	idon_L	L-Idonate
2	glcn	D-Gluconate
3	sbt_D	D-Sorbitol
4	mnl	D-Mannitol
5	5dglcn	5-Dehydro-D-gluconate
6	manglyc	2(alpha-D-Mannosyl)-D-glycerate
7	galt	Galactitol
8	gal_bD	beta D-Galactose
9	gal	D-Galactose
10	lyx_L	L-Lyxose
11	xylu_L	L-Xylulose
12	all_D	D-Allose
13	rib_D	D-Ribose
14	gam6p	D-Glucosamine 6-phosphate
15	xyl_D	D-Xylose
16	arab_L	L-Arabinose
17	gsn	Guanosine
18	man6p	D-Mannose 6-phosphate
19	f6p	D-fructose 6-phosphate
20	man	D-Mannose
21	g6p	D-Glucose 6-phosphate
22	fru	D-Fructose
23	psclys	psicoselysine
24	uri	Uridine
25	frulys	fructoselysine
26	xtsn	Xanthosine
27	gam	D-Glucosamine
28	melib	Melibiose
29	ins	Inosine
30	adn	Adenosine
31	gal1p	alpha-D-Galactose 1-phosphate
32	r5p	alpha-D-Ribose 5-phosphate
33	udpgal	UDPgalactose
34	acmana	N-Acetyl-D-mannosamine
35	acnam	N-Acetylneuraminate
36	acgam	N-Acetyl-D-glucosamine
37	g3pg	Glycerophosphoglycerol
38	malt	Maltose
39	acmum	N-Acetylmuramate
40	uacgam	UDP-N-acetyl-D-glucosamine
41	g1p	D-Glucose 1-phosphate
42	udpg	UDPglucose
43	malttr	Maltotriose
44	maltttr	Maltotetraose
45	sucr	Sucrose
46	maltpt	Maltopentaose
47	14glucan	1,4-alpha-D-glucan
48	malthx	Maltohexaose
49	lcts	Lactose
50	tre	Trehalose

Supplementary Table 2. The 41 original environments from which the *E. coli* metabolic network can adapt to at least 20 new single-carbon source environments

Number	Abbreviaiton	Full name
1	2ddglcn	2-Dehydro-3-deoxy-D-gluconate
2	5dglcn	5-Dehydro-D-gluconate
3	acgam	N-Acetyl-D-glucosamine
4	acmana	N-Acetyl-D-mannosamine
5	all_D	D-Allose
6	arab_L	L-Arabinose
7	ascb_L	L-Ascorbate
8	dha	Dihydroxyacetone
9	f6p	D-fructose 6-phosphate
10	fru	D-Fructose
11	frulys	Fructoselysine
12	fruur	D-Fructuronate
13	g1p	D-Glucose 1-phosphate
14	g6p	D-Glucose 6-phosphate
15	gal1p	alpha-D-Galactose 1-phosphate
16	gal	D-Galactose
17	gal_bD	beta D-Galactose
18	galctn_D	D-Galactonate
19	galctn_L	L-Galactonate
20	galt	Galactitol
21	galur	D-Galacturonate
22	gam6p	D-Glucosamine 6-phosphate
23	gam	D-Glucosamine
24	glc	D-Glucose
25	glcn	D-Gluconate
26	glcur	D-Glucuronate
27	glyald	D-Glyceraldehyde
28	glyc3p	Glycerol 3-phosphate
29	idon_L	L-Idonate
30	lyx_L	L-Lyxose
31	man6p	D-Mannose 6-phosphate
32	man	D-Mannose
33	manglyc	2(alpha-D-Mannosyl)-D-glycerate
34	mnl	D-Mannitol
35	psclys	Psicoselysine
36	r5p	alpha-D-Ribose 5-phosphate
37	rib_D	D-Ribose
38	sbt_D	D-Sorbitol
39	uri	Uridine
40	xyl_D	D-Xylose
41	xylu_L	L-Xylulose
